# Improving Perioperative Care in Gastric Surgery: Insights from the EUropean PErioperative MEdical Networking (EUPEMEN) Project

**DOI:** 10.3390/jcm14062108

**Published:** 2025-03-19

**Authors:** Orestis Ioannidis, Elissavet Anestiadou, Jose M. Ramirez, Nicolò Fabbri, Javier Martínez Ubieto, Carlo Vittorio Feo, Antonio Pesce, Kristyna Rosetzka, Antonio Arroyo, Petr Kocián, Luis Sánchez-Guillén, Ana Pascual Bellosta, Adam Whitley, Alejandro Bona Enguita, Marta Teresa-Fernandéz, Stefanos Bitsianis, Savvas Symeonidis

**Affiliations:** 1 Fourth Department of Surgery, Faculty of Health Sciences, Medical School, Aristotle University of Thessaloniki, General Hospital “George Papanikolaou”, 57010 Thessaloniki, Greece; elissavetxatz@gmail.com (E.A.); sbitsiani@gmail.com (S.B.); simeonidissavvas@yahoo.com (S.S.); 2 Institute for Health Research Aragón, 50009 Zaragoza, Spain; jramirez@unizar.es (J.M.R.); jmte-zubieto@hotmail.com (J.M.U.); anapascual689@gmail.com (A.P.B.); secretariagerm@gmail.com (A.B.E.); mteresa@iisaragon.es (M.T.-F.); 3 Department of Surgery, Faculty of Medicine, University of Zaragoza, 50009 Zaragoza, Spain; 4 Department of Surgery, Azienda Unità Sanitaria Locale Ferrara—University of Ferrara, 44121 Ferrara, Italy; n.fabbri@ausl.fe.it (N.F.); cvfeo@unife.it (C.V.F.); antonio.pesce@ausl.fe.it (A.P.); 5 Department of Anesthesia, Resuscitation and Pain Therapy, Miguel Servet University Hospital, 50009 Zaragoza, Spain; 6 Department of Plastic Surgery, Second Faculty of Medicine, Charles University and Motol University Hospital, 150 06 Prague, Czech Republic; kristina.rosetzua@gmail.com; 7 Department of Surgery, Universidad Miguel Hernández Elche, Hospital General Universitario Elche, 03203 Elche, Spain; arroyocir@hotmail.com (A.A.); drsanchezguillen@gmail.com (L.S.-G.); 8 Grupo Español de Rehabilitación Multimodal (GERM), 50009 Zaragoza, Spain; 9 Department of Surgery, Second Faculty of Medicine, Charles University and Motol University Hospital, 150 06 Prague, Czech Republic; kocian.cz@gmail.com; 10 Department of Surgery, University Hospital Kralovske Vinohrady, 100 34 Prague, Czech Republic; whitley.adam@gmail.com

**Keywords:** gastric surgery, perioperative care, enhanced recovery, EUPEMEN protocol, multidisciplinary collaboration

## Abstract

**Background/Objectives:** Gastric cancer remains a leading cause of cancer-related deaths worldwide and surgical resection represents the mainstay of treatment procedures. However, despite the advancements noted in the field of surgical oncology, perioperative complications and variability in the perioperative care provided persist. To address the challenges caused by non-standardized perioperative care for gastric surgery across European healthcare systems, the EUropean PErioperative MEdical Networking (EUPEMEN) protocol has been developed. The present study concisely provides the EUPEMEN protocol’s development, implementation, and impact on perioperative management in gastric resections. **Methods:** The EUPEMEN protocol was developed through a multidisciplinary collaboration involving five academic healthcare professionals from four European countries. The main activities of the collaborative group included a literature review, consensus development, the creation of multimodal rehabilitation manuals, and the development of an online learning platform. The EUPEMEN project aims for the uniform adoption of evidence-based practices across preoperative, intraoperative, and postoperative phases, leading in nutritional, psychological, and physiological optimization. **Results:** The implementation of the EUPEMEN protocol aims to optimize perioperative outcomes, including reduced postoperative complications, a shorter length of hospitalization, and improved recovery trajectories. The above have been achieved through structured guidelines that ensure consistent care delivery across diverse healthcare settings and tools such as rehabilitation manuals and a free-access online educational platform. **Conclusions:** The EUPEMEN protocol represents a new standard for perioperative care in the field of gastric surgery that is based on multidisciplinary collaboration and evidence-based practices. While challenges such as resource constraints and variability in adherence remain, the protocol demonstrates significant potential to improve patient outcomes and streamline perioperative management. Future research should focus on long-term effects and adaptation challenges in the setting of non-European healthcare systems.

## 1. Introduction

Gastric cancer is the fifth most common type of malignancy and the fourth most frequent cause of malignancy-related death worldwide [1]. Surgical resection in negative margins with lymphadenectomy remains the standard of care for localized gastric adenocarcinoma [2]. Despite advances in surgical oncology and especially in the field of minimally invasive surgical resections, gastric surgery has been associated with significant morbidity and 30-day mortality rates, ranging from 18% to 55% and from 2% to 8%, respectively, as well as prolonged recovery due to the complexity of the procedure and its impact on gastrointestinal function [3]. Postoperative complications, including delayed gastric emptying, anastomotic leakage, surgical site infections, and nutritional deficiencies, further complicate recovery and increase healthcare costs. Consequently, there is a pressing need for standardized, evidence-based perioperative care tailored to the specific needs of patients undergoing gastric surgery [4].

The enhanced recovery after surgery (ERAS) perioperative care pathway has revolutionized perioperative patient management by promoting evidence-based strategies to reduce surgical stress, improve recovery process, and minimize postoperative complications [5]. While the ERAS initiative has been widely adopted for various surgical procedures and has been successfully incorporated into perioperative planning of different gastrointestinal oncology surgeries, its application in gastric surgery still remains an important research question and the subject of numerous clinical trials and studies, mainly due to the unique challenges and difficulties associated with managing this patient population [6]. In addition, while ERAS protocols provide valuable frameworks for operative management, they often lack detailed guidance for day-to-day management and they do not specify and address the roles of every member of the multidisciplinary team providing perioperative care. This can lead to variability in implementation in clinical practice, particularly in complex surgical cases where precise team coordination and individualized care are crucial. Addressing these gaps is crucial to fully utilize the full potential of enhanced recovery pathways and ensure consistent, high-quality results.

Based on the foundational principles of ERAS, the EUPEMEN protocol provides evidence-based recommendations specifically designed for seven discrete modules of general surgery, among them also gastric surgery [7]. This collaboration resulted in the development of a standardized, open-access educational ERAS protocol, accessible via an online learning platform, which aims to promote the consistent implementation of evidence-based surgical practices in European healthcare facilities. 

This study presents the principles of EUPEMEN protocol for gastric surgery, outlining its key components, methodology, and day-to-day implementation strategies. By focusing on the integration of multidisciplinary care and tailored interventions, the protocol aims to promote and establish a new standard for perioperative management in gastric surgery, enhancing recovery while minimizing complications and resource utilization.

## 2. Materials and Methods

### 2.1. Protocol Development

The EUPEMEN project has been developed through the collaborative efforts of five academic partners with a strong clinical background from university hospitals across four European countries: Fundación Instituto de Investigación Sanitaria Aragón (Spain), Azienda Unità Sanitaria Locale Ferrara—University of Ferrara (Italy), Charles University and Motol University Hospital (Czech Republic), Universidad Miguel Hernández de Elche (Spain), and Papanikolaou General Hospital of Thessaloniki (Greece) [8].

### 2.2. Consensus Formation

A multidisciplinary European consensus panel conducted a targeted review of the literature on perioperative care in specific fields of general surgery, focusing on evidence-based practices to optimize recovery outcomes. An extensive literature review was conducted in PubMed, Scopus, and Cochrane Library using keywords such as “gastric surgery”, “perioperative care”, and “enhanced recovery”. Studies included were limited to English-language peer-reviewed publications from the past 15 years. The search strategy adhered to PRISMA guidelines, ensuring a rigorous and transparent selection of high-quality sources [9]. Key recommendations were developed and distributed among collaborators for refinements and additional input. This iterative process involved several rounds of anonymous surveys, followed by structured consensus meetings where disagreements were resolved, and the protocol was finalized. Each intervention included in the protocol was selected based on the strength of existing evidence, assessed using the GRADE framework. Preference was given to interventions with strong clinical trial support and meta-analyses confirming efficacy.

The protocol was developed through a three-phase Delphi process involving surgeons, anesthetists, nutritionists, and physiotherapists from multiple institutions [10]. Consensus was achieved through iterative rounds of anonymous surveys and structured discussions. A threshold of 75% agreement among participants was required for the inclusion of specific recommendations, ensuring a balanced and evidence-driven approach. The final consensus document underwent an external validation phase with experts reviewing the feasibility and applicability of the proposed measures.

### 2.3. Technical Activities

The EUPEMEN project involved six key technical activities designed to facilitate the development, dissemination, and implementation of the protocol [11]:

1. Development of multimodal rehabilitation manuals: Detailed manuals were created to provide comprehensive guidance on perioperative care for gastric cancer surgery and six other surgical disciplines, including esophageal, colorectal, liver, and bariatric surgery, as well as two commonly encountered conditions, including acute appendicitis and bowel obstruction. These manuals were translated into five languages (English, Spanish, Italian, Greek, and Czech) to ensure accessibility across Europe and made available through the EUPEMEN project website (https://eupemen.eu/eupemen-manuals/, accessed on 1 January 2025).
2. Creation of an online learning platform: A user-friendly online platform, accessible free of charge through the EUPEMEN Learning Website (https://eupemen.eu/, accessed on 1 January 2025), was established to host evidence-based, standardized perioperative protocols. This platform offers healthcare professionals free access to learning resources, interactive modules, and practical tools to enhance recovery outcomes in surgical patients.
3. Training future educators: A “train the trainer” approach was employed to ensure the sustainability of the project. Selected professionals were trained in the principles of enhanced recovery and equipped with the knowledge and skills to educate and mentor colleagues in their institutions.
4. Project promotion through multiplier events: Five large-scale promotional events were organized across participating countries to disseminate the EUPEMEN protocol, raise awareness of its benefits, and encourage widespread adoption in clinical practice.
5. International collaboration meetings: Four transnational meetings were held, bringing together experts from the participating institutions to refine the protocol, share experiences, and address challenges in implementation. These meetings facilitated consensus-building and strengthened the collaborative framework of the project.
6. Revision and standardization of protocols: The English version of the Recovery Intensification for Optimal Care in Adult Surgery (RICA) protocol was revised and adapted to create a unified set of perioperative care guidelines [12]. This process ensured that the EUPEMEN protocols aligned with the latest evidence and were tailored for effective application across diverse healthcare settings.

These technical activities were instrumental in establishing a robust framework for the EUPEMEN protocol, enabling its successful implementation and long-term impact.

### 2.4. Protocol Structure

The finalized EUPEMEN protocol for gastric resection is divided into three main phases:

- Preoperative phase: This phase emphasized comprehensive patient assessment, including nutritional evaluation, risk stratification, and psychological support. Multidisciplinary teams comprising surgeons, anesthesiologists, nurses, and dietitians worked collaboratively to optimize patient readiness through preoperative education, carbohydrate loading, and prophylactic measures to reduce surgical site infections and thromboembolic risks.
- Intraoperative phase: The intraoperative recommendations focused on minimizing surgical stress through techniques such as goal-directed fluid therapy, hemodynamic monitoring, and temperature control. Multimodal analgesia strategies were employed to reduce opioid use, and minimally invasive surgical approaches were prioritized to accelerate recovery.
- Postoperative phase: The postoperative phase prioritized early mobilization, oral intake initiation, and thromboembolism prevention through a combination of low-molecular-weight heparin and mechanical prophylaxis. Pain management relied on opioid-sparing multimodal analgesia, and patients were actively engaged in recovery through structured respiratory and functional physiotherapy sessions.

**Figure 1:**
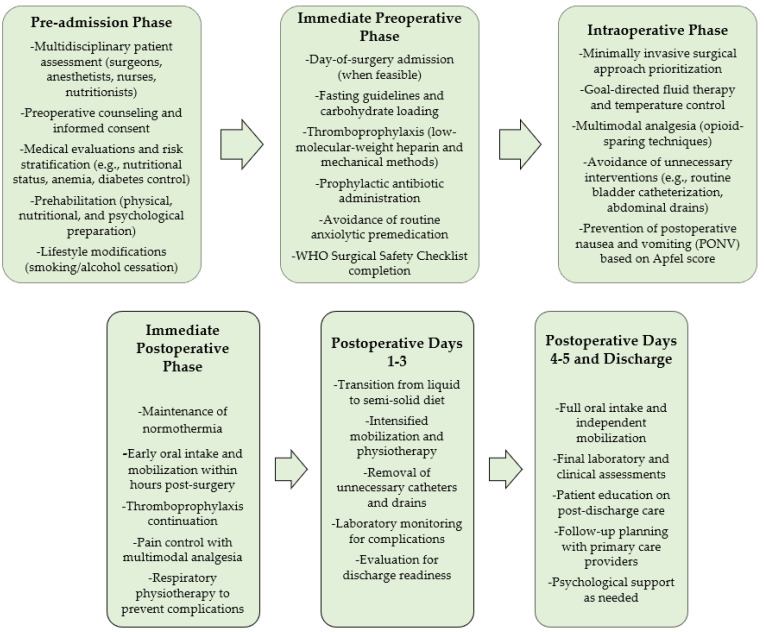
Phases of the EUPEMEN protocol for perioperative care in gastric surgery.

A summary of protocol phases is presented in Figure 1.

### 2.5. Objectives

The main goal of the EUPEMEN protocol is to establish a uniform approach to perioperative care in gastric surgery across European healthcare systems, reducing variability in practice and ensuring high-quality, evidence-based care. The specific goals include the following:

- Enhancing recovery by minimizing surgical stress and promoting early mobilization and oral intake.
- Reducing postoperative complications, such as infections, thromboembolic events, and delayed gastric emptying.
- Shortening hospital stays without compromising patient safety or outcomes.
- Providing a comprehensive framework that supports multidisciplinary collaboration, ensuring that all aspects of perioperative care are addressed effectively.
- Facilitating the education and training of healthcare professionals to implement and disseminate enhanced recovery protocols consistently.
- Establishing a unified standard of care that can be adapted to diverse healthcare settings, ultimately improving outcomes for gastric surgery patients.

### 2.6. Clinical Impact

The clinical impact of the EUPEMEN protocol is expected to be of the utmost importance, transforming the perioperative management of gastric surgery patients across Europe. By integrating evidence-based practices, the protocol aims to improve clinical outcomes, including reduced rates of surgical site infections, the faster return of gastrointestinal function, and improved pain control with minimal reliance on opioids. Early mobilization and nutritional optimization are anticipated to enhance functional recovery, enabling patients to resume normal activities sooner. In addition to direct patient benefits, the protocol is designed to have a systemic impact on healthcare delivery systems. Standardizing perioperative care will help to reduce hospital length of stay and healthcare costs, alleviating resource constraints in high-demand surgical units. Moreover, the multidisciplinary nature of the protocol fosters collaboration among healthcare professionals, improving communication and coordination within surgical teams. Finally, the EUPEMEN protocol contributes to the broader implementation of ERAS principles by addressing gaps in current guidelines, particularly the lack of detailed, procedure-specific recommendations for gastric surgery. This initiative not only improves patient outcomes but also serves as a model for future enhancements in perioperative care for other complex surgical procedures.

## 3. Results

The EUPEMEN Protocol for Gastric Surgery (Supplementary Materials).

### 3.1. Pre-Admission Phase

The pre-admission phase is a pivotal component of the perioperative pathway in gastric surgery, aiming to optimize patient outcomes and ensure procedural success [13]. This phase necessitates a multidisciplinary approach and is mainly performed by the collaboration of anesthetists, surgeons, nurses, and nutritionists to address the patient’s physical, nutritional, and psychological readiness for surgery [14].

Preoperative counseling plays a central role, where patients are thoroughly informed about the surgical procedure and the expected perioperative course. Taking into consideration that gastric resection has been associated with significant morbidity and mortality rates, providing data about 30-day mortality and short-term complications, as well as long-term outcomes, such as 90-day mortality, should be an integral part of the informed consent process [15]. This information is provided both verbally and in writing to ensure clarity and facilitate the patient’s informed consent, while also setting realistic expectations regarding postoperative outcomes to reduce preoperative anxiety [16]. A comprehensive medical assessment follows, encompassing a detailed medical history, physical examination, and essential diagnostic tests, including chest X-rays, blood analyses (coagulation parameters, biochemical and nutritional profiles, and full blood count), and electrocardiograms. These evaluations are crucial for identifying potential risks and tailoring the perioperative plan accordingly [17]. Chronic conditions must be carefully optimized before surgery. Patients with restrictive lung disease undergo spirometry, while those presenting significant cardiovascular risks, recent cardiovascular events, or a cardiovascular risk factor greater than 3, should be referred for specialized cardiology evaluation [18,19].

Anemia, especially iron deficiency anemia (IDA), presents a high prevalence in patients undergoing gastric resection, mainly due to gastric cancer, and requires proactive management, typically through parenteral iron administration to minimize perioperative complications [20]. An analysis of 834 patients by Peng et al. [21] revealed significantly worse disease-free survival rates in patients with type 2 diabetes mellitus compared to euglycemic individuals. To optimize perioperative outcomes, blood glucose and hemoglobin A1c (HbA1c) levels are assessed to ensure the adequate control of diabetes mellitus [22]. Poorly controlled or previously undiagnosed diabetes necessitates timely intervention by primary care or endocrinology specialists [23].

Up to 85% of patients with gastric cancer are diagnosed with poor nutritional status preoperatively. Malnutrition has been associated with increased morbidity and mortality rates, an increased length of hospital stay, and poorer adjuvant treatment tolerance and response [24]. Preoperative nutritional status is another critical factor that determines perioperative results after gastric resection [25]. Among nutritional screening tools used, the Malnutrition Universal Screening Tool (MUST) score is often used for cancer patients and is proposed for identifying patients at risk of malnutrition [26]. Preoperative nutritional deficiencies and insufficiencies, such as those involving calcium, iron, vitamin D, and B12, found at screening, are corrected to enhance surgical recovery [27,28,29]. For patients experiencing aphagia or solid dysphagia, individualized dietary interventions, including alternative routes of administration of artificial nutrition, liquid diets, and high-caloric protein supplements, are implemented [30]. Lifestyle modifications, particularly the cessation of tobacco use and the reduction of alcohol consumption, are encouraged as soon as a surgical diagnosis is established, given their profound impact on perioperative and long-term outcomes [31,32].

It has been proven that multimodal prehabilitation, including the actions performed to improve patients’ physical, nutritional, and emotional status preoperatively, plays a beneficial role in the functional abilities of frail elderly patients, through the reduction of chronic inflammation and oxidative stress and improved lipid metabolism [33,34]. Equally important to physical preparation, the psychological readiness of the patient should also be prioritized. Patients are offered counseling to address any mental health concerns, which can significantly influence recovery trajectories [35,36]. In addition, tailored physical exercises focusing on cardiovascular, respiratory, and muscular strengthening further enhance the patient’s physiological resilience [37,38]. Risk assessment tools such as the Apfel scoring system for anticipating postoperative nausea and vomiting (PONV) and identifying patients in need of PONV prophylaxis, as well as the ASA score for overall perioperative risk stratification, are routinely employed as part of the preoperative anesthesiologic assessment to inform clinical decision-making and individualized care plans [39,40].

Collectively, these pre-admission measures provide a structured and comprehensive approach, ensuring that patients are well-prepared for the challenges of gastric surgery. This meticulous preparation not only mitigates perioperative risks but also sets the stage for improved postoperative recovery and long-term outcomes.

### 3.2. Perioperative Phase

#### 3.2.1. Immediate Preoperative Phase

The immediate preoperative phase in gastric surgery represents a critical window for the final preparations to ensure patient safety and optimize surgical outcomes [41,42]. It involves specific interventions designed to minimize perioperative risks, performed collaboratively by surgeons, anesthetists, and nursing staff. Ideally, patients are admitted on the day of surgery to reduce unnecessary hospitalization, provided that logistical and clinical considerations allow [43].

Fasting protocols are an essential component of this phase. Preoperative fasting aims to enable sufficient time for the emptying of gastric content and, thus, minimizes the possibility of pulmonary aspiration during anesthesia [44]. Patients are advised to abstain from solid food for at least eight hours prior to surgery and to cease the intake of clear liquids two hours before anesthesia induction [45,46]. This approach minimizes the risk of aspiration during the procedure while adhering to enhanced recovery guidelines. In addition, this preoperative fasting protocol has been associated with decreased organic response to trauma and inflammation and reduced insulin resistance while avoiding the undesired symptoms of thirst, hunger, anxiety, and malaise and limiting patient dissatisfaction [45]. Furthermore, the administration of a preoperative dose of low-molecular-weight heparin as a form of thromboprophylaxis should be scheduled 2–12 h before surgery, tailored to the planned use of neuraxial anesthesia and the patient’s thromboembolic risk [47]. This intervention is complemented by mechanical thromboprophylaxis measures, such as compression stockings or intermittent pneumatic compression devices [47,48].

Further preparation includes prophylactic measures to reduce the risk of postoperative complications. A single carbohydrate-rich drink (12.5% maltodextrins, 400 mL) is administered two hours before anesthesia induction, barring contraindications [49]. This practice has been shown to reduce insulin resistance and inflammatory response and enhance recovery [50]. For patients with delayed gastric emptying, apart from appropriate fasting, additional measures are taken to prevent regurgitation during induction, including antacid prophylaxis, mechanical gastric emptying, appropriate patient positioning, and the careful selection of anesthetic technique and maneuvers from induction to emergence [51]. Prophylactic antibiotics are administered within 30–60 min of the surgical incision, selected based on local microbial resistance patterns, in order to lower the risk of surgical site infections [52,53].

Minimizing unnecessary interventions preoperatively is equally important. Routine preoperative anxiolytic premedication is avoided and should be discouraged, as it can interfere with early postoperative mobilization and physical and cognitive recovery. Literature reports have associated anxiolytic premedication with a negative overall effect on the perioperative patient experience due to serious side effects, such as paradoxical reactions, oversedation, and airway obstruction [54].

Similarly, hair removal at the surgical site of interest, if necessary, is performed using an electric razor to reduce skin trauma and subsequent infection risk [55]. These small but significant details align with evidence-based practices to streamline preoperative care and support patient outcomes.

In overall, the immediate preoperative phase embodies a balance between thorough preparation and minimizing invasiveness. Through the implementation of these structured protocols, this stage ensures that patients enter the operating room in the best possible condition, paving the way for successful surgery and recovery.

#### 3.2.2. Intraoperative Phase

The intraoperative phase represents a cornerstone of perioperative management in gastric surgery, ensuring patient safety, optimizing surgical outcomes, and minimizing intra- and postoperative complications. During this phase, a multidisciplinary approach involving surgeons, anesthetists, and nursing staff is essential to deliver seamless care.

A critical first step in the intraoperative phase is the completion of the World Health Organization (WHO) Surgical Safety Checklist prior to the surgical incision, aiming to improve patient safety. This checklist serves as a safeguard, ensuring that all procedural elements are accounted for and potential risks are proactively addressed [56]. Continuous intraoperative monitoring is performed throughout the procedure, including the assessment of vital signs, the fraction of inspired oxygen (FiO_2_), and the consideration of the depth of anesthesia, the neuromuscular blockade, and glycemic levels [57,58,59,60]. Additionally, non-invasive hemodynamic monitoring is recommended to maintain optimal physiological parameters and to reduce the need for invasive measures [61].

Invasive procedures such as arterial or central venous catheter placement are generally avoided unless clinically indicated, such as in patients with severe cardiorespiratory compromise or elevated risks of postoperative renal failure [62]. Routine bladder catheterization is also omitted in uncomplicated cases, reducing the risk of catheter-associated urinary tract infections and enhancing postoperative comfort [63]. Anesthesia is managed with short-acting agents for both induction and maintenance, facilitating swift recovery and minimizing residual effects [64]. Oxygenation is carefully maintained with an FiO_2_ exceeding 50% to mitigate the risk of hypoxemia [65].

Optimal fluid therapy during this phase is pivotal to maintaining hemodynamic stability and adequate organ and tissue perfusion, and is one of the key elements of enhanced recovery after surgery protocols [66]. Goal-directed fluid management using validated devices is recommended, but when unavailable, a restrictive approach tailored to the patient’s ideal body weight is implemented [67,68]. Perioperative normothermia is of paramount importance for achieving reduced blood loss and lower surgical site infection as well as a lower overall complication rate [69,70]. Currently, maintaining normothermia is a priority, achieved through active warming techniques such as heated infusions and thermal blankets, thereby mitigating complications associated with intraoperative hypothermia [71].

PONV is commonly encountered in high-risk patients after laparoscopic gastrointestinal surgery due to the maneuvers and resection of the stomach or bowel [72]. Prophylactic measures against PONV are administered based on the Apfel risk scoring system, targeting high-risk individuals with appropriate antiemetic therapy [73]. Effective pain management is pivotal in patients after open and laparoscopic gastric resection and multimodal analgesia, combining analgesic regimen with different mechanisms of action, and it aims to fully exert analgesic effects while reducing the risk of adverse effects [74]. Analgesia strategies are tailored to the surgical approach; thoracic epidural analgesia is preferred for open procedures, while alternatives such as transverse abdominis plane blocks are employed in laparoscopic cases or when epidural use is contraindicated, as well as in patients at risk for postoperative renal failure or coagulopathy [75,76,77].

Laparoscopic gastrectomy presents favorable outcomes, including reduced perioperative blood loss, faster postoperative recovery, and fewer complications, while providing oncological adequate regarding lymph node harvesting and resection margins [78]. Thus, minimally invasive surgical approach is prioritized wherever feasible, reflecting the best practices for reducing surgical trauma and enhancing postoperative recovery. The use of nasogastric tubes is limited to intraoperative gastric decompression; there is no convincing evidence to support routine drain use after gastrectomy, and thus, abdominal drains should be avoided to reduce infection risks and postoperative discomfort [79,80]. 

This structured and evidence-based intraoperative framework not only safeguards patients during surgery but also lays the foundation for enhanced postoperative recovery, aligning with contemporary principles of enhanced recovery after surgery (ERAS).

#### 3.2.3. Immediate Postoperative Phase

The immediate postoperative phase in gastric surgery is a pivotal period focused on stabilizing the patient, preventing complications, and initiating recovery. This phase is managed collaboratively by anesthetists and nursing staff to ensure a smooth transition to postoperative care and enhanced recovery protocols.

Normothermia in the postoperative period has been associated with a decreased incidence of infectious complications and a shorter length of hospital stay [81]. Maintaining normothermia is a priority, achieved through regular monitoring and the use of active warming methods, such as heated blankets or warmed intravenous fluids [82]. Hypothermia, a frequent concern after surgery, is effectively mitigated through these interventions, supporting better postoperative outcomes. Gastrointestinal surgery, especially for oncologic indications, results in great physical trauma, severe postoperative acute pain, and strong stress response, leading to decreased immune function, an increased risk of a localized tumor or distant recurrence [83]. As a consequence, effective pain management is the central aim during this phase, emphasizing opioid-sparing multimodal analgesia [84]. By targeting a pain score of less than 3 on the visual analog scale (VAS), where 0 represents “no pain” and 10 represents “worst possible pain”, this approach minimizes the adverse effects associated with opioids while providing adequate pain relief [85].

Early oral intake after gastrectomy is encouraged within six hours post-surgery, provided the patient is clinically stable. This practice presents several advantages over bowel rest and intravenous nutrition, such as the activation of normal digestive reflexes and decreased patient discomfort and anxiety, while the preservation of the immune system promotes wound healing and increases resistance to infections [86]. In the same orientation, early mobilization is initiated promptly, with patients being advised to sit upright within three hours and ambulate within six hours, except during nighttime hours [87]. Early mobilization plays a vital role in improving recovery and reducing the risks of complications, such as venous thromboembolism, without increasing readmission rate or the severity of postoperative complications [88].

To prevent thromboembolic events, thromboprophylaxis is a critical component of care, as the reported rate of venous thromboembolism in patients with gastric cancer ranges from 5.3% to 25.5% [89]. The combination of pharmacological prophylaxis with low-molecular-weight heparin (LMWH) administered 12 h after surgery, and mechanical methods such as compression stockings, has been proven as an effective approach towards the prevention of thromboembolic events [90]. PONV may be encountered in up to 30% of the total of patients and up to 70% of “high-risk” in patients during the postoperative phase and should be managed using antiemetic therapies tailored to the patient’s risk profile, as determined by the Apfel score [91]. This targeted approach ensures effective symptom control, supporting faster progression to oral intake and mobility.

By implementing these evidence-based practices, the immediate postoperative phase is optimized to enhance recovery and minimize complications. This approach reflects modern ERAS principles, ensuring a high standard of care and improved outcomes for patients undergoing gastric surgery [4].

### 3.3. Postoperative Day 1

On the first postoperative day, patient management transitions from immediate postoperative care to functional recovery under the coordinated efforts of the surgical team and nursing staff. An early liquid diet has been proven to be a feasible and safe approach and may lead to a shorter length of hospital stay [92,93]. A liquid diet is initiated based on the patient’s tolerance to oral intake with the cessation of intravenous fluid therapy and should be adopted as a standard for perioperative care after gastric surgery [86]. Early mobilization is prioritized as a basic element of enhanced recovery after surgery protocol, as it enhances gastrointestinal recovery, with encouragement to ambulate and engage in light activities [94]. A strategy of opioid-sparing multimodal analgesia is employed, aiming for a VAS score below 3 [95]. Intravenous fluids are discontinued once the patient achieves adequate oral hydration [96]. Low-molecular-weight heparin is administered to mitigate thromboembolic risks, alongside the use of compression stockings or intermittent pneumatic compression.

Adverse events such as urinary tract infection or inadequate patient mobilization may be prevented by early catheter removal [97]. Surgical and nursing staff assess the suitability for removing urinary catheters or abdominal drains where applicable. Postoperative pulmonary complications (PPC) rank among the major causes affecting the prognosis of patients after gastrectomy for gastric malignancies and burdening healthcare costs [98]. Intensive respiratory physiotherapy, supported by physiotherapists, should be introduced to prevent PPC.

### 3.4. Postoperative Day 2

On the second postoperative day, the patient progresses to a semi-solid diet, guided by the surgeon and nutritionist, and early mobilization activities are intensified under the supervision of the surgeon and physiotherapy team [86]. Pain control strategies continue to prioritize minimizing opioid use, targeting a VAS score of less than 3. For patients with epidural catheters, coagulation status must be meticulously assessed to ensure hemostatic adequacy prior to removal and the procedure should confirm the complete and intact retrieval of the catheter to mitigate the risk of hematoma or catheter fragment retention [99]. Physiotherapists implement targeted respiratory and functional exercises, and nurses assist in monitoring for any early signs of complications. Last but not least, the thromboprophylaxis scheme should be maintained as per protocol.

### 3.5. Postoperative Day 3

The third postoperative day involves preparing the patient for potential discharge, managed collaboratively by the surgeon, nurses, and physiotherapists. The patient transitions to a soft diet under the guidance of the surgeon and nutritionist [100]. Active mobilization is encouraged, with sustained periods of ambulation emphasized. Pain management relies on active or preventive multimodal, opioid-sparing analgesia, aiming for a VAS score below 3, while thromboprophylaxis continues to reduce the risk of thromboembolic events. Nurses and surgeons oversee laboratory evaluations, including C-reactive protein, procalcitonin, and a full blood count, to monitor recovery and detect anastomotic leakage, bleeding, and major infective complications [101]. The multidisciplinary team evaluates discharge readiness, ensuring the patient meets the criteria for safety and functionality [102]. More particularly, discharge should be considered if the patient demonstrates no surgical complications, remains afebrile, has pain controlled with oral analgesia, achieves full ambulation, tolerates oral food intake, and consents to discharge.

### 3.6. Postoperative Day 4

On the fourth postoperative day, the focus is on consolidating recovery and ensuring readiness for discharge. A bland diet is provided to maintain gastrointestinal comfort while meeting nutritional requirements. Patients are expected to ambulate independently and actively participate in respiratory and functional physiotherapy. Thromboprophylaxis continues as per protocol, and laboratory tests are repeated to monitor and detect any latent complications. Discharge criteria are re-evaluated, and patients who meet all clinical and functional benchmarks should be considered for discharge with appropriate post-discharge care instructions. This structured approach ensures optimal recovery and minimizes postoperative risks.

### 3.7. Discharge

Discharge planning focuses on ensuring patient safety and continuity of care. The discharge phase involves surgeons, nurses, dietitians, physiotherapists, and stoma therapists (if applicable), collaboratively ensuring recovery readiness, providing tailored instructions, and coordinating follow-up care. Criteria for discharge include the absence of surgical complications, no signs of infection or fever, effective pain control with oral analgesia, the ability to ambulate independently, and the adequate tolerance of oral food intake [103]. Before discharge, patients should be provided with comprehensive documentation outlining their medical status and detailed recommendations for continued care. This includes nutritional guidance tailored to their recovery, any prescribed medications, and instructions for monitoring their health. Additionally, patients are educated on recognizing signs of complications and are encouraged to seek medical attention promptly if needed. Comprehensive instructions are provided to the patient regarding wound care, activity levels, and follow-up appointments. Nutritional needs are evaluated with caloric, protein, mineral, and vitamin intake assessment according to patient needs and are addressed with specific dietary recommendations [104]. Telephone follow-up and coordination with primary care or home support services is facilitated to ensure a smooth transition to recovery at home. The patient’s psychological well-being and quality of life should also be considered, with referrals to psychology specialists when needed, ensuring a holistic approach to postoperative recovery [105].

## 4. Discussion

Enhanced recovery after surgery (ERAS) programs have revolutionized perioperative care in a substantial way by introducing evidence-based, multimodal strategies aimed at minimizing the physiological stress of surgery, reducing postoperative complications, and accelerating patient recovery [106]. First introduced by Henrik Kehlet in the 1990s in order to address multimodal perioperative morbidity, these programs emphasize a holistic approach, integrating interventions across the preoperative, intraoperative, and postoperative phases of care [107]. Key elements include preoperative patient education, optimized nutrition, opioid-sparing analgesia, early mobilization, and streamlined discharge planning. ERAS protocols have shown positive effects in patient outcomes, including reduced hospital stays, lower complication rates, and enhanced patient satisfaction [108].

While ERAS programs have gained widespread acceptance in elective procedures such as colorectal and bariatric surgery, their adoption in complex or resource-intensive surgeries, like gastric procedures, has been slower and remains an evolving process due to the unique physiological and oncological challenges associated with these procedures [109]. In addition, the clinical benefits of the ERAS pathway are still under debate in gastric cancer surgery [110]. The implementation of the ERAS protocol resulted in a significantly lower incidence of anastomotic leakage and a significant reduction in the median length of hospital stay, compared to historical data [111]. A recent single-center retrospective study by Liu et al. [112], in 2025, examined the short- and long-term outcomes of ERAS management in patients undergoing minimally invasive radical gastrectomy after neoadjuvant chemotherapy. The patients of the ERAS group presented significantly lower levels of novel inflammatory biomarkers, improved nutritional status, and faster postoperative recovery, as well as a trend toward an increased 3-year overall survival, compared to patients of the traditional group [112]. A narrative review by Rosa et al. highlighted that while ERAS pathways improve short-term outcomes in gastric cancer patients, the variability in protocol components among institutions impedes uniform adoption [6]. Apart from clinical factors, circumstances related to healthcare systems and the socio-cultural characteristics of the patients as well as diversity in surgical procedures have been associated with impaired adoption of ERAS gastric cancer programs [113]. While these guidelines provide a broad framework, they often lack procedural specificity, particularly regarding nutritional optimization and multidisciplinary collaboration.

Challenges such as variability in implementation, resource limitations, and resistance to change underscore the need for tailored and standardized protocols, such as the EUPEMEN initiative, to expand the reach and impact of enhanced recovery programs across diverse surgical disciplines and healthcare settings. The EUPEMEN project, a multi-center European initiative supported by EU funding, was developed with the primary goal of educating future generations of healthcare professionals who will serve as trainers [8]. The EUPEMEN protocol is grounded in evidence-based perioperative principles derived from ERAS guidelines while incorporating additional refinements to address the specific needs of gastric surgery patients. A comprehensive literature review was conducted, evaluating studies from PubMed, Scopus, and Cochrane Library to identify gaps in perioperative care. The review highlighted inconsistencies in nutritional optimization, multimodal analgesia application, and postoperative recovery strategies, necessitating the development of a structured and standardized approach. Furthermore, key findings from clinical trials and meta-analyses on perioperative management in gastric surgery were integrated to refine the protocol. The EUPEMEN protocol, therefore, serves as a framework for harmonizing perioperative care by implementing standardized, multidisciplinary interventions across various European healthcare systems.

The project aims to promote and expand the adoption of ERAS recommendations, enhancing the integration and effectiveness of enhanced recovery programs in routine clinical practice. Additionally, the protocol emphasizes adaptability, ensuring that its principles can be applied across different healthcare infrastructures while maintaining the core ERAS principles. More particularly, the EUPEMEN protocol for gastric surgery reflects a significant advancement in the standardization of perioperative care, tailored to the unique challenges associated with gastric cancer treatment. While ERAS protocols have demonstrated improvements in postoperative outcomes across various surgical disciplines, their application to gastric surgery has historically been less developed. The EUPEMEN initiative for gastric surgery addresses this gap by integrating evidence-based practices into a structured framework designed specifically for gastric procedures. A major strength of the EUPEMEN protocol is its focus on multidisciplinary collaboration and harmonization across European healthcare systems. The inclusion of surgeons, anesthesiologists, dietitians, nurses, physiotherapists, and other healthcare professionals ensures a comprehensive approach that addresses every aspect of the patient’s perioperative care [114]. This collaboration enables tailored interventions, such as nutritional optimization, multimodal analgesia, and early mobilization, all of which are critical for improving recovery outcomes in gastric surgery. Another notable feature of the protocol is its adaptability. By providing resources such as open-access multimodal rehabilitation manuals and an online learning platform, EUPEMEN facilitates widespread adoption and local customization. These tools not only standardize care but also address variability in clinical practices, a common challenge in implementing ERAS principles [115]. The train-the-trainer approach further enhances the protocol’s sustainability by equipping professionals to disseminate knowledge within their institutions. The EUPEMEN initiative builds upon ERAS models by incorporating digital learning resources and real-time implementation monitoring, ensuring sustained adherence and quality improvement across diverse healthcare settings.

The clinical impact of the EUPEMEN protocol is substantial. By reducing surgical stress, enhancing recovery, and minimizing complications, the protocol improves patient outcomes while potentially reducing the length of stay at the hospital and healthcare costs. Moreover, the structured guidelines promote consistency in care delivery, ensuring that patients across diverse healthcare settings benefit from high-quality, evidence-based practices.

While ERAS has significantly contributed to standardizing perioperative care and improving patient outcomes, its implementation in gastric surgery has faced specific challenges [116]. The EUPEMEN protocol was developed to address these limitations by refining the framework with a greater emphasis on multidisciplinary coordination, structured nutritional optimization, individualized risk stratification, and enhanced rehabilitation strategies. One of the primary shortcomings of ERAS in gastric surgery is its limited specificity in perioperative interventions tailored to this patient population [117]. Gastric cancer patients often present with unique physiological challenges, including malnutrition, altered metabolic status, and increased perioperative stress responses, which are not always comprehensively addressed in existing ERAS guidelines. Furthermore, ERAS lacks detailed guidance on the multidisciplinary roles of surgeons, anesthetists, nutritionists, and physiotherapists in optimizing recovery. The EUPEMEN protocol, by contrast, prioritizes a highly structured, team-based approach that ensures every phase of perioperative care is managed collaboratively to reduce variability in clinical practice. Another key limitation of traditional ERAS pathways is the inconsistency in the application of nutritional interventions. Many ERAS programs acknowledge the importance of preoperative nutritional status but do not provide specific screening methodologies, intervention thresholds, or individualized supplementation strategies [118]. The EUPEMEN protocol integrates detailed preoperative nutritional risk assessment tools, including the Malnutrition Universal Screening Tool (MUST) and specific nutritional supplementation regimens, to ensure that patients are adequately prepared for surgery. This is particularly relevant for gastric cancer patients, who frequently suffer from cachexia, micronutrient deficiencies, and reduced physiological reserves, making nutritional optimization a cornerstone of perioperative success.

Postoperative rehabilitation is another area where the EUPEMEN protocol expands upon existing ERAS models [119]. While ERAS promotes early mobilization and opioid-sparing analgesia, the EUPEMEN protocol goes further by incorporating structured physiotherapy regimens and standardized pain management algorithms tailored to gastric surgery patients. In addition, real-time adherence monitoring and digital learning platforms have been developed under EUPEMEN to ensure consistent implementation across different healthcare settings, a component often lacking in ERAS protocols.

Moreover, other perioperative protocols, such as those developed within institution-specific or national guidelines, often lack harmonization across different healthcare systems. While some institutions may have individualized enhanced recovery pathways, these are typically heterogeneous in structure and lack a universally accepted framework for multidisciplinary collaboration. The EUPEMEN protocol bridges this gap by providing a standardized, evidence-based framework applicable across European healthcare systems, ensuring greater uniformity in perioperative care.

Lastly, EUPEMEN is designed to be highly adaptable and integrates a training-of-trainers approach, enabling healthcare professionals to disseminate knowledge and improve adoption rates across multiple institutions. This structured educational component ensures that ERAS principles are not only implemented but also sustained through continued professional development, a crucial factor often overlooked in perioperative care standardization.

Finally, it should be highlighted that the EUPEMEN protocol is designed to be adaptable across all stages of gastric cancer, ensuring comprehensive perioperative management regardless of disease progression. While the fundamental principles remain consistent, additional considerations are integrated for patients with advanced-stage disease. These patients often present with higher rates of malnutrition, reduced functional capacity, and increased perioperative risk, necessitating intensified prehabilitation measures, individualized nutritional optimization, and extended postoperative monitoring [120]. By incorporating stratified perioperative interventions, the EUPEMEN protocol ensures that all gastric cancer patients receive tailored care aligned with their specific clinical needs.

## 5. Future Research Directions

While the EUPEMEN protocol has been developed to standardize perioperative care in gastric surgery across European healthcare systems, further research is needed to evaluate its long-term impact on patient outcomes, healthcare resource utilization, and cost-effectiveness. Future studies should focus on longitudinal follow-ups to assess postoperative recovery trajectories, late complications, and overall survival rates in patients managed under this protocol. Additionally, evaluating patient-reported outcomes and quality of life measures will provide a more comprehensive understanding of the protocol’s benefits beyond clinical parameters. Moreover, future studies should include structured evaluations of physicians’ perspectives and experiences with the EUPEMEN protocol. Understanding their feedback, barriers to implementation, and perceived benefits will be critical in refining and optimizing the protocol for broader clinical adoption.

Given the variability in healthcare infrastructure, surgical expertise, and perioperative care practices globally, adapting the EUPEMEN protocol for non-European settings represents a crucial next step. Collaborative efforts should aim to assess the feasibility of implementation in diverse healthcare environments, particularly in low- and middle-income countries where resource constraints may pose challenges. Identifying key modifications to accommodate different healthcare systems while maintaining the core principles of standardized, evidence-based perioperative care will be essential for ensuring the broader applicability of this initiative.

Furthermore, integrating digital tools such as real-time data monitoring, telemedicine support, and artificial intelligence-driven analytics may enhance protocol adherence and enable personalized perioperative management strategies. Future research should explore how these innovations can optimize outcomes and streamline perioperative workflows within the EUPEMEN framework.

## 6. Challenges and Limitations

The implementation of the EUPEMEN protocol across diverse healthcare settings presents several challenges that must be considered for its widespread adoption. Variability in institutional resources, including access to specialized perioperative care teams and advanced monitoring tools, may affect the feasibility of full adherence to the protocol, necessitating adaptable implementation strategies. Differences in pre-existing perioperative practices and institutional cultures may also influence protocol compliance, highlighting the need for structured training programs and continuous education to facilitate seamless integration. Additionally, resistance to change among healthcare professionals remains a potential barrier, emphasizing the importance of demonstrating tangible clinical benefits through pilot studies and real-world evidence. To address these challenges, a phased implementation approach incorporating continuous feedback mechanisms, digital adherence monitoring tools, and institutional engagement strategies is recommended to ensure a smooth and effective transition to standardized perioperative care in gastric surgery.

It should be underlined that, while the EUPEMEN protocol represents a significant advancement in perioperative care, certain limitations warrant consideration. First, the absence of long-term clinical data restricts the ability to comprehensively evaluate its impact on patient outcomes, healthcare costs, and overall EUPEMEN protocol’s efficacy beyond the immediate postoperative period. Second, the protocol’s reliance on resources, such as advanced perioperative monitoring equipment and multidisciplinary teams, may limit its applicability in under-resourced settings. Institutions with constrained budgets or staffing shortages may face challenges in achieving full adherence to the protocol, potentially compromising its effectiveness. Third, variability in compliance across institutions and healthcare systems presents a significant obstacle. Differences in cultural attitudes, organizational structures, and professional practices can affect the uniformity of protocol implementation, as seen in other ERAS programs. Addressing these disparities will require targeted interventions, including education, auditing, and feedback mechanisms. Lastly, the focus on a European healthcare context may limit the protocol’s generalizability to other regions with differing healthcare systems, patient populations, and disease burdens. Adaptations for non-European settings should also be explored to broaden the protocol’s applicability and relevance.

Despite these limitations, the EUPEMEN protocol establishes a robust framework for enhancing perioperative care. Addressing these challenges through research, resource allocation, and stakeholder engagement will be essential to fully realize its potential.

Currently, no pilot data are available to quantitatively substantiate the expected benefits of the EUPEMEN protocol. While projections based on literature could provide theoretical estimations, relying on such data without direct clinical validation carries a risk of bias and the potential misrepresentation of real-world outcomes. Recognizing this limitation, we emphasize the necessity of future prospective studies to rigorously assess the protocol’s impact on perioperative outcomes, complication rates, and hospital length of stay. A multi-center evaluation framework is being developed to systematically collect and analyze empirical data, ensuring that future findings will provide robust evidence to support the protocol’s efficacy. These results, once available, will be disseminated in subsequent publications to further refine and optimize perioperative management strategies within gastric surgery.

## 7. Conclusions

In conclusion, the EUPEMEN protocol represents a paradigm shift in the perioperative management of gastric surgery, setting a new standard for patient-centered care and offering a model for broader application in other surgical disciplines.

## Data Availability

The data presented in this study are available at https://eupemen.eu/. accessed on 1 January 2025.

## Supplementary Materials

The following supporting information can be downloaded at https://www.mdpi.com/article/10.3390/jcm14062108/s1: Eupemen Protocol.

## Abbreviations

The following abbreviations are used in this manuscript:

ASA	American Society of Anesthesiologists
ERAS	Enhanced Recovery After Surgery
EUPEMEN	European Perioperative Medical Networking
FiO2	Fraction of Inspired Oxygen
HbA1c	Hemoglobin A1c
LMWH	Low-Molecular-Weight Heparin
MUST	Malnutrition Universal Screening Tool
PONV	Postoperative Nausea and Vomiting
PPC	Postoperative Pulmonary Complications
RICA	Recovery Intensification for Optimal Care in Adult Surgery
VAS	Visual Analog Scale
WHO	World Health Organization
