# Supplementary material for: Improving Perioperative Care in Gastric Surgery: Insights from the EUropean PErioperative MEdical Networking (EUPEMEN) Project
Source: J Clin Med. 2025 Mar 19;14(6):2108. doi: 10.3390/jcm14062108 (PMC11942800; doi:10.3390/jcm14062108)
Supplement: Supplementary file 1 [file jcm-14-02108-s001.zip › jcm-3493976-supplementary.pdf]

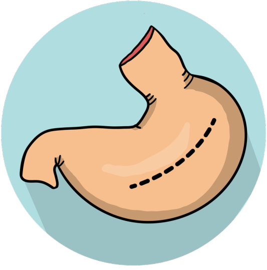

EUPEMEN PROTOCOL

GASTRIC RESECTION

1	Before Admission<br><br>Anaesthetist, Surgeon, Nurse, Nutritionist
1.1	**Preoperative counselling**<br>The patient should be fully informed on procedure and perioperative course both verbally and in writing. Signed informed consent should be obtained.
1.2	**Comprehensive medical assessment**<br>This should include medical history, physical examination, chest X-ray, blood tests (coagulation parameters, biochemical profile, nutritional profile and full blood count) and electrocardiogram.
1.3	**Compensation of chronic diseases**<br>All chronic diseases should be optimised before surgery. Preoperative spirometry should be performed for patients with restrictive lung disease. Cardiology evaluation if cardiovascular risk factor is greater than 3. All cases of recent onset or active cardiovascular diseases should be evaluated by a cardiologist.
1.4	**Evaluation and treatment of iron deficiency and preoperative anaemia**<br>Iron deficiency anaemia should be ideally managed by parenteral iron administration.
1.5	**Evaluation of diabetes mellitus**<br>Blood glucose and HbA1c levels should be investigated. All cases of poorly controlled or previously undiagnosed diabetes should be referred to primary care or endocrinology before surgery.
1.6	**Nutritional optimisation**<br>Assess nutritional status with the MUST test. Correct preoperative nutritional deficiencies including calcium, iron, vitamin D and vitamin B12. For patients with aphagia assess potential routes of administration of artificial nutrition. For solid dysphagia give liquid diets with high-caloric protein supplements.
1.7	**Abandon tobacco use and reduce alcohol consumption**<br>Use of tobacco should be stopped and alcohol consumption should be reduced as soon as diagnosis is made.
1.8	**Physical exercises**<br>Cardiovascular, respiratory and muscle strengthening exercises tailored to the physical state of the patient.
1.9	**Psychological counselling**<br>Any psychological issues the patient may be facing should be fully addressed.
1.10	**Apfel score**<br>The risk for postoperative nausea and vomiting should be assessment with the Apfel score.
1.11	**ASA assessment**

EUPEMEN European Perioperative Medical Networking
Co-funded by the Erasmus+ Programme of the European Union
Cátedra GERM Medicina Perioperatoria Universidad Zaragoza

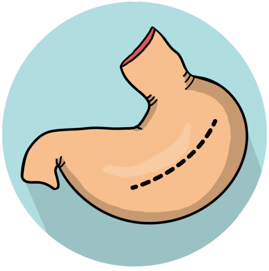

	As part of the preoperative anaesthesiologic assessment the ASA score should be obtained.
2	Perioperative
2.1	Immediate Preoperative<br>(Schedule admission the same day of surgery if possible)<br><br>Anaesthetist, Surgeon, Nurse
2.1.1	**Preoperative fasting**<br>Patients should not be allowed to eat solid food 8 hours before the surgery and drink liquids 2 hours before the surgery.
2.1.2	**Low molecular weight heparin**<br>Low molecular weight heparin should be administered 2-12 hours before surgery (depending on whether neuraxial anaesthesia is to be performed or not).
2.1.3	**Compression stockings**<br>Placement of compression stockings or intermittent pneumatic compression, according to thromboembolic risk.
2.1.4	**Carbohydrate drink**<br>A single carbohydrate drink (12.5% maltodextrins) 400 ml should be given 2 hours prior to anaesthesia if there is no contraindication.
2.1.5	**Avoid anxiolytic premedication**<br>Do not add preoperative anxiolytic premedication.
2.1.6	**Shaving with electric razor**<br>The site where the incision will be performed should be shaved with an electric razor, if necessary.
2.1.7	**Antibiotic prophylaxis**<br>Antibiotic prophylaxis should be given 30-60 min before surgical incision. The choice of antibiotic should be made based on local hospital protocol.
2.1.8	**Prophylactic measures for prevention of gastric regurgitation**<br>For patients with delayed gastric emptying prophylactic measures should be made to prevent regurgitation.
2.2	Intraoperative<br><br>Anaesthetist, Surgeon, Nurse
2.2.1	**WHO Surgical Safety Checklist**<br>The WHO Surgical Safety Checklist should be completed before incision is made.
2.2.2	**Routine intraoperative monitoring**

EUPEMEN European Perioperative Medical Networking

Co-funded by the Erasmus+ Programme of the European Union

Cátedra GERM Medicina Perioperatoria Universidad Zaragoza

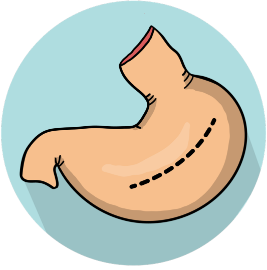

EUPEMEN PROTOCOL

GASTRIC RESECTION

	Vital functions, FiO2, anaesthesia depth with, neuromuscular blockade and glycemia should be monitored during the procedure. Non-invasive hemodynamic monitoring is also recommended.
2.2.3	**Avoid arterial catheters**<br>Invasive arterial catheter is not routinely required. Although it should be used for patients with severe cardiorespiratory disorders.
2.2.4	**Avoid central venous catheters**<br>Central venous catheters are not routinely required for minor resections and in the absence of risk factors for postoperative renal failure.
2.2.5	**Avoid routine bladder catheterization**
2.2.6	**Induction and maintenance of anaesthesia**<br>Short-acting agents should be used for induction and maintenance of anaesthesia.
2.2.7	**Oxygenation**<br>Patients should receive oxygen with a FiO2 of more than 50%.
2.2.8	**Fluid therapy**<br>Hemodynamic optimization using goal-guided fluid therapy with validated devices is recommended. If these are not available, restrictive fluid therapy is recommended based on ideal weight.
2.2.9	**Prevention of hypothermia**<br>Temperature should be monitored and normothermia should be maintained by active heating (heated infusions, heated blanket).
2.2.10	**Prophylaxis of postoperative nausea and vomiting**<br>Give antiemetic therapy according to the Apfel score.
2.2.11	**Epidural analgesia**<br>Thoracic epidural analgesia should be used in open surgery. In laparoscopic surgery it is not routinely recommended. Patients with contraindication for epidural analgesia and who have a risk for postoperative renal failure or coagulopathy could benefit from bilateral transverse abdominis plane block or other alternatives to epidural analgesia.
2.2.12	**Minimally invasive surgery**<br>Minimally invasive approaches are preferred and should be used as much as possible.
2.2.13	**Avoid nasogastric tubes**<br>Nasogastric tubes are only recommended intraoperatively in order to empty the stomach.
2.2.14	**Avoid abdominal drains**

EUPEMEN
European Perioperative Medical Networking

Co-funded by the
Erasmus+ Programme
of the European Union

Cátedra GERM
Medicina Perioperatoria
Universidad Zaragoza

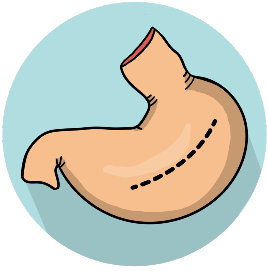

EUPEMEN PROTOCOL

GASTRIC RESECTION

**2.3**	**Immediate Postoperative**<br>Anaesthetist, Nurse
2.3.1	**Maintenance of normothermia**<br>Temperature should be regularly measured and normothermia should be maintained.
2.3.2	**Opioid sparing analgesia**<br>Active or preventive multimodal analgesia should be used. Restrict the use of opioids. Aim for a VAS score of less than 3.
2.3.3	**Early feeding**<br>Beginning of oral fluid intake from 6 hours after the surgery.
2.3.4	**Early mobilisation**<br>Mobilization should begin 3 hours after surgery and should begin with sitting up in bed. Ambulation should begin 6 hours after surgery with respect to night time hours for sleeping.
2.3.5	**Thromboembolic prophylaxis**<br>Low Molecular Weight Heparin should be given 12 hours after surgery.
2.3.6	**Prophylaxis of postoperative nausea and vomiting**<br>Give antiemetic therapy according to the Apfel score.
**3**	**Postoperative Day 1**<br>(Ward)<br>Surgeon, Nurse
3.1	**Early feeding**<br>A liquid diet should be started, according to patient tolerance.
3.2	**Early mobilization**<br>Patients should be encouraged to walk.
3.3	**Opioid sparing analgesia**<br>Active or preventive multimodal analgesia should be used. Restrict the use of opioids. Aim for a VAS score of less than 3.
3.4	**Stop intravenous fluids**<br>If patients tolerate adequate peroral liquid intake stop intravenous fluid therapy.
3.5	**Remove urinary catheter**<br>Evaluate withdrawal of urinary catheter if one is in place.
3.6	**Removal of abdominal drains**<br>If a drain has been placed assess its removal.
3.7	**Thromboprophylaxis**

EUPEMEN European Perioperative Medical Networking
Co-funded by the Erasmus+ Programme of the European Union
Cátedra GERM Medicina Perioperatoria Universidad Zaragoza

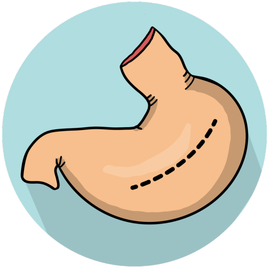

EUPEMEN PROTOCOL

GASTRIC RESECTION

3.8	**Respiratory physiotherapy**
4	Postoperative Day 2<br>(Ward)<br><br>Surgeon, Nurse
4.1	**Early feeding**<br>Give patients semi-solid diets (purees, yoghurts, etc.).
4.2	**Early mobilization**<br>Patients should be encouraged to walk.
4.3	**Opioid sparing analgesia**<br>Active or preventive multimodal analgesia should be used. Restrict the use opioids. Aim for a VAS score of less than 3.
4.4	**Remove epidural catheter**<br>Remove epidural catheter if it has been placed. First control coagulation parameters and make sure the entire catheter is removed.
4.5	**Respiratory and functional physiotherapy**
4.6	**Thromboprophylaxis**
5	Postoperative Day 3<br>(Ward)<br><br>Surgeon, Nurse
5.1	**Early feeding**<br>Soft diet.
5.2	**Early mobilization**<br>Patients should be encouraged to walk.
5.3	**Opioid sparing analgesia**<br>Active or preventive multimodal analgesia should be used. Restrict the use opioids. Aim for a VAS score of less than 3.
5.4	**Respiratory and functional physiotherapy**
5.5	**Thromboprophylaxis**
5.6	**Laboratory blood tests**<br>These should include C-reactive protein, procalcitonin and full blood count.
5.7	**Discharge**

EUPEMEN European Perioperative Medical Networking
Co-funded by the Erasmus+ Programme of the European Union
Cátedra GERM Medicina Perioperatoria Universidad Zaragoza

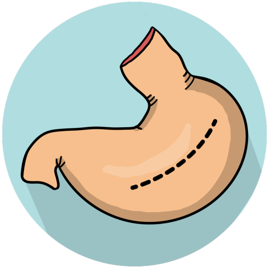

EUPEMEN PROTOCOL

GASTRIC RESECTION

	Consider discharge if: no surgical complications, no fever, pain controlled with oral analgesia, full ambulation, tolerance of oral food intake and if the patient agrees.
6	Postoperative day 4<br>(Ward)<br><br>Surgeon, Nurse
6.1	**Early feeding**<br>Bland diet.
6.2	**Early mobilization**<br>Patients should be encouraged to walk.
6.3	**Opioid sparing analgesia**<br>Active or preventive multimodal analgesia should be used. Restrict the use opioids. Aim for a VAS score of less than 3.
6.4	**Respiratory and functional physiotherapy**
6.5	**Thromboprophylaxis**
6.6	**Laboratory blood tests**<br>These should include C-reactive protein, procalcitonin and full blood count.
6.7	**Discharge**<br>Consider discharge if: no surgical complications, no fever, pain controlled with oral analgesia, full ambulation, tolerance of oral food intake and if the patient agrees.
7	Discharge<br><br>Surgeon, Nurse, Psychologist, Primary care
7.1	**Patient documentation**<br>Provide patients with information of medical state and give recommendations for further care.
7.2	**Continued care**<br>Telephone control after discharge. Home support coordination with primary care
7.3	**Nutritional state.**<br>Assess caloric, protein, mineral and vitamin intake according to needs.
7.4	**Psychology consultation**<br>Refer patients to psychology specialists if necessary. Assess postoperative quality of life.

EUPEMEN European Perioperative Medical Networking
Co-funded by the Erasmus+ Programme of the European Union
Cátedra GERM Medicina Perioperatoria Universidad Zaragoza
